# Heart failure with reduced ejection fraction developed from valvular surgery: Risk factors and therapeutic effects of sacubitril valsartan

**DOI:** 10.1016/j.ijcha.2025.101634

**Published:** 2025-02-25

**Authors:** Chen Yuqian, Hu Qinghua, Luo Fanyan, Huang Lingjin, Chen Xuliang, Zhang Chengliang

**Affiliations:** a Department of Cardiovascular Surgery, Xiangya Hospital of Central South University, Changsha, Hunan Province, China; b Graduate School of Central South University, Changsha, Hunan Province, China

**Keywords:** Cardiac valve surgery, Heart failure with reduced ejection fraction, Risk factors, ARNI

## Abstract

**Objective:**

To investigate the risk factors for heart failure developed from cardiac valvular surgery and the efficacy of sacubitril valsartan (ARNI).

**Methods:**

Clinical data from patients with left ventricular ejection fraction (LVEF) ≥ 50 % who consecutively underwent cardiac valvular (mitral/aortic valve) surgery in 2021 were collected. Pre − and intra − operative variables were analyzed to explore risk factors for HFrEF (LVEF ≤ 40 %). Post − operative HFrEF patients were split into ARNI − treated (n = 15) and non-ARNI − treated (n = 15) groups. Follow − up echocardiography data were compared to assess ARNI’s efficacy.

**Results:**

Among 420 patients undergoing valve surgery (117 aortic, 133 mitral, 170 double-valve), 34 (8.1 %) developed HFrEF, showing significantly higher in-hospital mortality than non-HFrEF patients (8.82 % vs 0.52 %). Multivariate analysis identified preoperative left ventricular diameter as an independent HFrEF risk factor. During follow-up, 70 % of HFrEF patients achieved LVEF > 50 % within 6 months, Repeated-measures F test demonstrated significantly greater LVEF improvement (P = 0.036) and LVEDD reduction (P = 0.014) in the ARNI group versus non-ARNI group.

**Conclusions:**

About 8 % of patients with LVEF ≥ 50 % developed HFrEF after cardiac valvular surgery, and large left ventricular diameter was an independent risk factor. Sacubitril valsartan is very effective in improving left ventricular remodeling and LVEF in such cohort.

## 1 Introduction

Heart failure with reduced ejection fraction (HFrEF), which is diagnosed when left ventricular ejection fraction (LVEF), measured by echocardiography, is 40 % or lower, is a major public health concern with substantial morbidity and mortality. Although the management of HFrEF has remarkably improved in recent decades, its mortality remains high, with a 5-year survival rate of 25 % [1], [2]. The majority of the available studies on HFrEF have focused on chronic heart failure among the general population. Few studies have focused on HFrEF developed from cardiac surgery.Table 1.

**Table 1:** Nonstandard Abbreviations and Acronyms.

Abbreviation	Full name/Meaning
LVEDD	Left ventricular end-diastolic dimension
LAD	Left atrial diameter
LVEF	Left ventricular ejection fraction
LVFS	Left Ventricular Fractional Shortening
TTE	Transthoracic echocardiography
Ang II	Angiotensin II receptor
ARNI	Angiotensin receptor neprilysin inhibitor
ACEI	Angiotensin-converting-enzyme inhibitors
ARB	Angiotensin receptor blockers
MRA	Mineralocorticoid Receptor Antagonist
SLGT2 inhibitors	Sodium-glucose cotransporter 2 inhibitors
Preserved LVEF	LVEF greater than 50 % but with elevated natriuretic peptide levels or diastolic dysfunction

In recent years, in an increasingly aged society, the age of patients with cardiac valvular disease has been increasing. Nevertheless, the surgical mortality related to valvular heart disease has stabilized at less than 2 %[3].In our clinical practice, we have noticed that a good number of patients survived cardiac valve surgery but living with a significant decrease in LVEF or even HFrEF. Kammerlander and colleagues reported that 12 % patients had HF with mid-range or reduced ejection fraction after left-sided valve surgery[4]. In addition to increasing the morbidity and mortality, the HFrEF prevalence led to longer patient recovery periods at intensive care units (ICU) and increased consumption of medical resources. Previous studies, including the STS and Euro score system, have uncovered the risk factors for death in cardiac valvular surgery[5], [6]. Nevertheless, the risk factors predisposing to HFrEF after surgery remain unknown.

It is well known that sacubitril valsartan as the first dual inhibitor of Ang II receptor and neprilysin is very effective in treating HFrEF. Sacubitril valsartan can reduce mortality and rehospitalization rate, and remarkably improve left ventricular remodeling and function when used in HFrEF patients[7], [8]. However, at present, clinical study on the efficacy of sacubitril valsartan in cardiac surgery-related heart failure is very scarce, and we thus focused on sacubitril valsartan’s effects in this cohort.

The main purpose of this study is to explore the potential risk factors for HFrEF developed from cardiac valve surgery and get to know its’ short-term prognosis. Our second goal is to evaluate the clinical efficacy of sacubitril-valsartan in such cardiac surgery-related heart failure patients.

## 2 Subjects and methods

### 2.1 Inclusion criteria

1:patients who underwent cardiac valve replacement at the Department of Cardiovascular Surgery, Xiangya Hospital of Central South University (Changsha, China), from January 1 to December 31, 2021, were collected. 2:preoperative LVEF ≥ 50 %. 3:Patients who are self-aware and can express themselves clearly. 4:Patients requiring valvular surgery were clearly diagnosed by preoperative imaging. 5:The same group of doctors performed valvular surgery through median thoracotomy. 6:Patients with a follow-up time of at least 6 month and complete follow-up data.

### 2.2 Exclusion criteria

1: preoperative LVEF < 50 %.2: combined valve surgery in which the primary surgery was a coronary artery bypass grafting (CABG), ascending aorta or aortic arch surgery, or hypertrophic obstructive cardiomyopathy. 3: independent tricuspid valve surgery.4: minimally invasive valve surgery.5: Emergency operation. 6: patients with malignant tumors or other systemic diseases.

### 2.3 Data collection

Clinical data from all enrolled patients were collected from the medical record database of the Xiangya Hospital. All data collection was conducted between March and May 2023.This study was approved and written informed consent was waived by the Ethics Committee of the Xiangya Hospital, Central South University (approval number: 202204103) due to it is retrospective nature. The preoperative variables included age, weight, gender, etiology, heart function classification, comorbidities, preoperative transthoracic echocardiography (TTE) data, as well as laboratory tests of the patients. The intraoperative data were involved surgical procedures, the cardio-pulmonary bypass（CPB）time, and the aortic clamping time. The post-operative outcomes including major adverse events, ICU stay, hospital stay, and echocardiography data were also collected. For patients with HFrEF developed from vavular surgery, information such as medication after discharge, heart function and TTE data at 1 month, 3 months, and 6 months after surgery was obtained.Authors had access to information that could identify individual participants after data collection.

### 2.4 Valvular surgical method

The surgical protocol has been previously described[9]. Briefly, the patient was subjected to a general anesthesia and placed in a supine position. A median sternotomy was performed. A CPB was performed through the cannulation of the ascending aorta and the superior and inferior vena cava. Right atrial cannulation was performed when isolated aortic valve was involved. Drainage of the left heart was done through the right upper pulmonary vein. The nasopharyngeal temperature was maintained between 32 and 34℃. Cardiac arrest was achieved by perfusing a modified Del Nido solution every 60 min, either through the aortic root or the orifice of the coronary arteries. The mitral valve replacement was performed via a *trans*-septal approach, while the aortic valve replacement was performed via a transverse aortotomy. In some patients, combined surgeries, such as CABG, left atrial folding, and modified maze procedure for atrial fibrillation, were performed. In each patient, an intraoperative transesophageal echocardiography (TEE) was used for monitoring and evaluation. All patients were admitted to the ICU immediately after surgery. Transthoracic echocardiography (TTE) was reexamined about 5 days after surgery or when necessary.

### 2.5 Groups

**Fig. 1:**
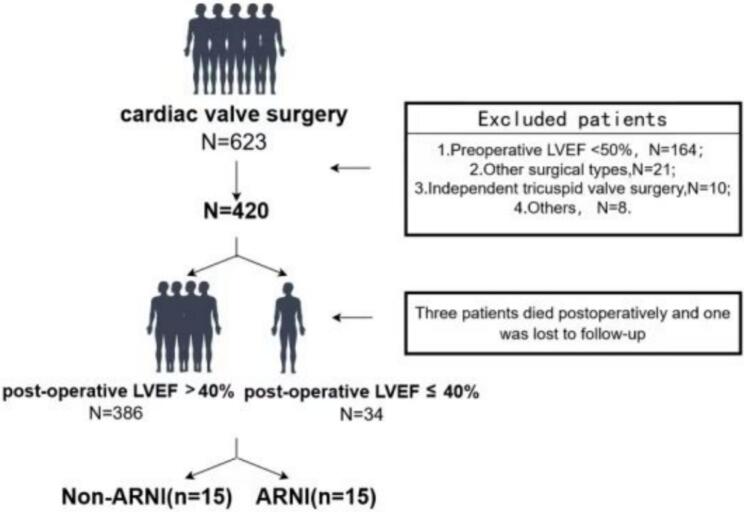
Flowchart.

In the present study, patients were diagnosed with HFrEF when the post-operative TTE showed LVEF was ≤ 40 %, while others with LVEF > 40 % were placed into the non-HFrEF group. Patients with HFrEF developed from vavular surgery were divided into the ARNI group or the Non-ARNI group according to they received sacubitril valsartan treatment or not (Fig. 1). As noticed, the starting dosage for most patients was 25–50 mg twice a day and it was gradually titrated to 50–100 mg twice a day. Of note, other heart failure drugs such as diuretics, beta blocker and mineralocorticoid receptor antagonist (MRA) were also routinely used in each group.

### 2.6 Statistical Methods

The SPSS software version 25. 0 (SPSS Inc, Chicago, IL) was used for statistical analysis. Continuous variables were expressed as mean ± standard deviation. Counting and categorical variables were expressed as case numbers (percentage). For continuous variables, an univariate analysis was performed using the independent samples *t*-test if it fits for Gaussian distribution or the Mann-Whitney *U* test if not. The chi-square test was used for the counting and categorical variables. A p-value ≤ 0.05 was considered statistically significant. To explore potential risk factors for developing HFrEF after surgery, all significant factors screened by the univariate analysis were substituted into the unconditional multivariate logistic regression analysis model. Since follow-up data were measured multiple times at different time points, repeated measurement analysis of variance was applied.

## 3 Results

### 3.1 General data

Among all patients who underwent cardiac valve surgery in the Xiangya Hospital （China）in 2021, 420 patients with preoperative LVEF ≥ 50 % were enrolled. The mean age in this cohort was 55.67 ± 10.37 years,with 50.7 % of the patients were male. According to the New York Heart Association(NYHA) classification,the majority of the patients were classified into gradeⅡ(21.7 %) or grade Ⅲ(61.7 %). The etiology of the valvular heart disease included rheumatic, degenerative, infective, and congenital conditions. Rheumatic heart disease accounted for 54.8 % (n = 230).Other information such as accompanying diseases, number of the involved heart valves and intraoperative factors were also detailed in Table 2.

**Table 2:** Univariate analysis for risk factors for HFrEF.

Variable	The non-HFrEF group（n = 386）	The HFrEF group（n = 34）	P value
Gender			
female	196（50.8 %）	11（32.4 %）	0.039*
male	190（49.2 %）	23（67.6 %）	
Age (year)	55.57 ± 10.25	56.76 ± 11.73	0.520
Weight (kilogram)	59.17 ± 10.88	61.15 ± 11.84	0.320
NYHA heart function			
Ⅰ	10（2.6 %）	0（0.0 %）	0.534
Ⅱ	104（26.9 %）	8（23.5 %）	
Ⅲ	238（61.7 %）	21（61.8 %）	
Ⅳ	34（8.8 %）	5（14.7 %）	
Number of involved valves			
single	127（32.9 %）	13（38.2 %）	0.302
double	187（48.4 %）	12（35.3 %）	
three	72（18.7 %）	9（26.5 %）	
Etiology			
rheumatic	215（55.7 %）	15 (44.1 %)	0.507
degenerative	85（22.0 %）	10 (29.4 %)	
infectious	57（14.8 %）	7 (20.6 %)	
congenital	29（7.5 %）	2（5.9 %）	
Valve and type of disease			
Mitral valve			
stenosis	93(33.4 %)	9(36 %)	0.678
insufficiency	95(34.2 %)	10(40 %)	
both	90(32.4 %)	6(24 %)	
Aortic valve			
stenosis	88(33.6 %)	7(28 %)	0.485
insufficiency	94(35.9 %)	12(48 %)	
both	80(30.5 %)	6(24 %)	
Diabetes mellitus	32（8.3 %）	5（14.7 %）	0.342
Hypertension	71（18.4 %）	12（35.3 %）	0.018*
Atrial fibrillation	119（30.8 %）	12（35.3 %）	0.590
Coronary artery disease	43（11.1 %）	9（26.5 %）	0.020*
Combined atrial folding	41（10.6 %）	7（20.6 %）	0.142
Combined CABG	27（7.0 %）	6（17.6 %）	0.060
Combined maze procedure	57（14.8 %）	5（14.7 %）	0.992
Hemoglobin (g/dl)	121.91 ± 22.19	118.56 ± 24.72	0.403
Serum creatinine (μmol/L)	93.62 ± 67.66	98.19 ± 42.70	0.699
Total bilirubin (μmol/L)	16.86 ± 12.42	18.19 ± 8.78	0.549
Albumin (g/dl)	37.49 ± 4.86	35.68 ± 4.94	0.038*
LVEF-pre（%）	60.69 ± 6.30	57.47 ± 5.88	0.004*
LVFS-pre(%)	33.07 ± 5.18	30.79 ± 4.32	0.013*
LVEDD-pre(mm)	53.49 ± 8.196	60.06 ± 9.53	0.000*
LAD-pre(mm)	48.47 ± 10.57	51.00 ± 8.811	0.176
CPB time-intra (min)	110.69 ± 44.69	132.94 ± 58.01	0.036*
Aortic clamping time-intra(min)	81.22 ± 34.17	102.24 ± 48.21	0.018*

(NYHA heart function::New york Heart Association heart function;GABG:Coronary Artery Bypass Grafting;LVEF-pre:Left ventricular ejection fraction-preoperative;LVFS-pre:Left Ventricular Fractional Shortening-preoperative;LVEDD-pre:Left ventricular end-diastolic dimension-preoperative;LAD-pre:Left atrial diameter-preoperative).

### 3.2 Surgical outcomes

There was no patient death during or immediately after surgery, and the overall in-hospital mortality rate was 1.2 % (5/420). Based on the results of post-operative TTE, patients were divided into the HFrEF group (LVEF: 33.07 ± 7.03 %，n = 34, 8.1 %) and the non-HFrEF group (LVEF: 50.27 ± 8.14 %，n = 386, 91.9 %). In the HFrEF group, 3 out of 34 patients died of heart failure, with a mortality rate of 8.82 %. In the non-HFrEF group, one patients died, due to multiple organ dysfunction syndrome and the other one died due to malignant arrhythmia and cardiac arrest, with a mortality rate of 0.052 %. Also, the duration of the hospital stay (19.62 ± 4.13 vs. 12.98 ± 3.22 days) and the ICU stay (5.31 ± 6.26 vs. 2.12 ± 4.35 days) were remarkably longer in the HFrEF group than in the non-HFrEF group. The number of newly onset postoperative atrial fibrillation (AF) was 32 (8.29 %) in the non-HFrEF group and 2 (6.88 %) in the HFrEF group, and there was no difference between the two groups.

### 3.3 Univariate analysis

In order to screen out possible risk factors for HFrEF developed from valvular surgery, all the collected preoperative and intraoperative variables between the HFrEF and the non-HFrEF groups were analyzed. Among the preoperative variables, the proportion of male patients and the incidences of hypertension or coronary artery diseases were higher in the HFrEF group than in the non-HFrEF group. The LVEF, as measured by preoperative TTE, was a slightly lower in the HFrEF group, while the left atrial and ventricular diameters were larger in the HFrEF group. Regarding the intraoperative variables, the duration of the CPB and the aortic clamping time were longer in the HFrEF group than in the non-HFrEF group. The proportion of combined CABG was higher in the HFrEF group (Table 2).

### 3.4 Multivariate regression analysis

After being screened out by the univariate analysis, potential variables including the gender, hypertension or coronary artery diseases, albumin levels, LVEDD and LAD, duration of the CPB and aortic clamping, and combined CABG, were substituted into a binary logistic regression model for further analysis. The results demonstrated that only LVEDD was significantly related to HFrEF development after surgery, with an odds ratio of 1.069 (95 % CI: 1.013–1.127, p-value = 0.014, Table 3).

**Table 3:** Outcomes of multivariate regression analysis.

variable	regression coefficient	standard error	Odd ratio	95 % confidence interval	P value
Gender	0.087	0.447	1.091	0.454–2.622	0.846
Coronary disease	0.603	0.594	1.827	0.570–5.859	0.310
Hypertension	0.741	0.416	2.098	0.929–4.741	0.075
LVEDD	0.067	0.027	1.069	1.013–1.127	0.014*
LVEF	0.052	0.206	1.053	0.703–1.578	0.800
LVFS	−0.018	0.280	0.836	0.483–1.446	0.521
CPB time	−0.014	0.012	0.986	0.964–1.008	0.213
Aortic clamping time	0.025	0.015	1.026	0.997–1.055	0.081
Albumin (g/dl)	−0.050	0.039	0.951	0.881–1.027	0.200
Combined CABG	0.076	0.705	1.079	0.271–4.297	0.914

(LVEDD:Left ventricular end-diastolic dimension;LVEF:Left ventricular ejection fraction;LVFS:Left Ventricular Fractional Shortening;CPB:Cardiopulmonary Bypass;CABG:Coronary Artery Bypass Grafting).

### 3.5 Efficacy of sacubitril-valsartan in patients with surgery-related HFrEF

A total of 31 patients with HFrEF developed from surgery were dischagerd and were closely followed up in the outpatient department. One patient in the Non-ARNI group was lost during the follow-up for unknown reason. The follow-up observation period in this study was 6 months. Independent samples T-test and chi-square test were used to compare the general data of the patients between the ARNI group (n = 15) and the Non-ARNI group (n = 15), and the results showed that there was no significant difference between the two groups in baseline general data (Table 4).

**Table 4:** General characteristics of patients in ARNI and Non-ARNI groups.

Variable	Number or mean ± standard deviation		
	ARNI group （n = 15）	Non-ARNI group（n = 15）	P value
Gender			
female	4	2	0.651
male	11	13	
Age (year)	55.80 ± 12.53	50.40 ± 15.51	0.174
BMI	22.49 ± 3.91	22.29 ± 3.34	0.734
Etiology			
rheumatic	7	8	0.559
degenerative	6	3	
infectious	1	1	
congenital	1	3	
Medical history			
diabetes mellitus	1	2	1
hypertension	5	3	0.682
atrial fibrillation	8	3	0.058
coronary artery disease	2	3	1
Postoperative medications			
MRA	14	13	0.543
SLGT2 inhibitors	1	2	0.543
Beta-blockers	5	4	0.690
Number of involved valves			
single	6	5	0.711
double	6	5	
three	3	5	
Combined surgery			
atrial folding	4	2	0.651
maze procedure	3	2	1
CABG	1	0	1
NYHA heart function-pre			
Ⅱ	1	5	0.145
Ⅲ	11	9	
Ⅳ	3	1	
LAD-Post（mm）	44.87 ± 5.33	42.47 ± 9.33	0.141
LVEDD-Post（mm）	58.53 ± 6.15	63.67 ± 8.16	0.245
LVEF-Post（mm）	34.67 ± 7.23	32.93 ± 9.48	0.400

(BMI:Body Mass Index;NYHA heart function-pre:New york Heart Association heart function-preoperative;LAD-Post:Left atrial diameter-postoperative；LVEDD-Post:Left ventricular end-diastolic dimension-postoperative;LVEF-Post:Left ventricular ejection fraction-postoperative).

**Fig. 2:**
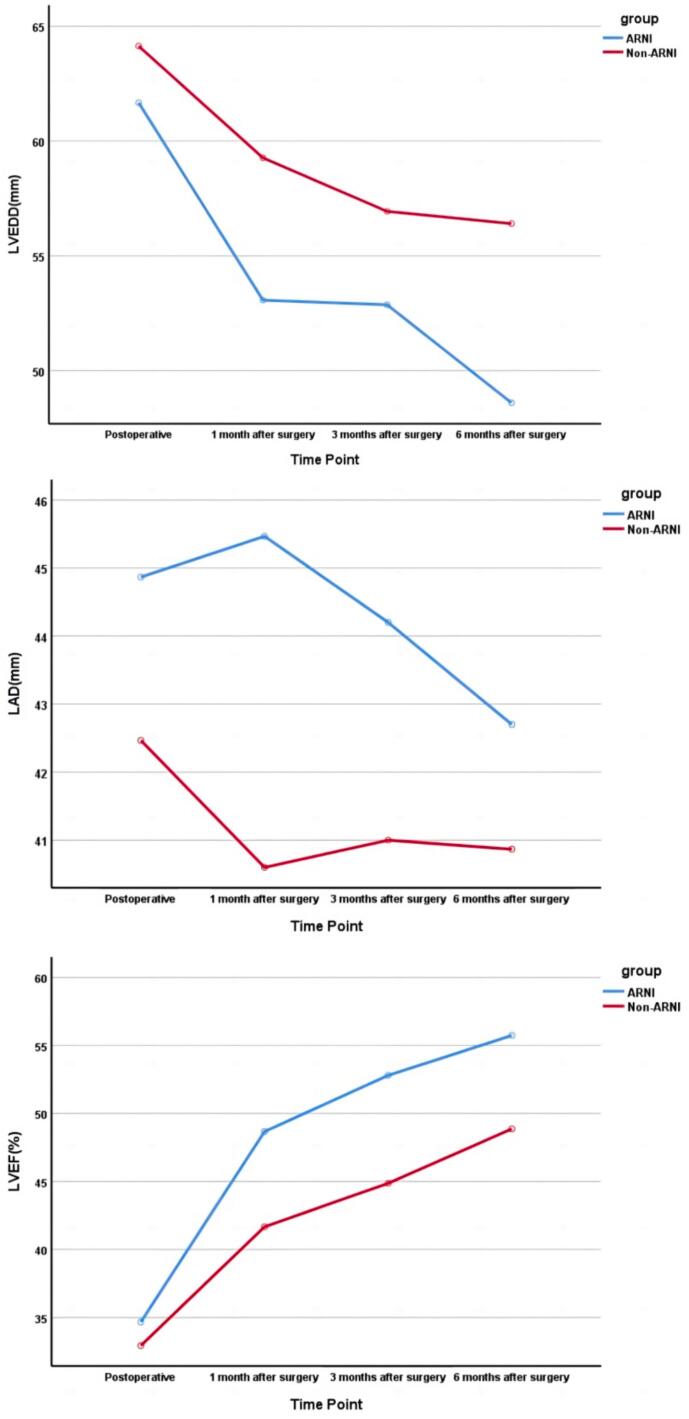
Changes of LVEDD, LAD and LVEF during follow-up.

The echocardiogram data of two groups of patients were collected at post − operation, 1 month, 3 months and 6 months after operation. During follow-up, the majority of the patients who developed HFrEF after cardiac valvular surgery recovered well after discharge. Except for reduced activity tolerance, there was no other sequalue in the reduced LVEF patients in our cohort, and the LVEF in 70 % of the patients was 50 % or greater within 6 months. Repeated measures F-test results showed that there were significant differences in the between − group main effects of left ventricular end − diastolic diameter (LVEDD) (F = 6.87; P = 0.014) and LVEF (F = 4.88*; P = 0.036), indicating that the overall performance of the ARNI group was significantly better than that of the Non − ARNI group. At the same time, there were also significant differences in the time main effects of LVEDD (F = 23.59; P = 0) and LVEF (F = 48.90; P = 0), suggesting that the LVEDD and LVEF in both groups changed significantly over time. While LAD did not show significant change either compared with baseline or between the two groups (Table 5 and Fig. 2). The results indicated that for patients with HFrEF after cardiac surgery, the use of ARNI was effective in reducing LVEDD and increasing LVEF.

**Table 5:** Follow-up echocardiography data and Repeated measures F-test results.

Echocardiography data		Baseline	1 mo post − op	3 mo post − op	6 mo post − op	Repeated measures F-test	
		M ± SD	M ± SD	M ± SD	M ± SD	F valve	P valve

LVEDD	ARNI	61.67 ± 6.11	53.07 ± 7.0	52.87 ± 7.21	48.60 ± 6.87		
	Non-ARNI	64.13 ± 7.36	59.27 ± 6.04	56.93 ± 7.83	56.40 ± 6.64		
	Group main effect					6.87	0.014*
	Time main effect					23.59	0*
	Group*Time					1.63	0.21
LAD	ARNI	44.87 ± 5.33	45.47 ± 9.03	44.20 ± 9.35	42.70 ± 9.47		
	Non-ARNI	42.47 ± 9.33	40.6 ± 7.38	41.00 ± 8.24	40.87 ± 8.08		
	Group main effect					1.30	0.264
	Time main effect					0.89	0.42
	Group*Time					0.62	0.55
LVEF	ARNI	34.67 ± 7.23	48.67 ± 12.35	52.8 ± 7.35	55.73 ± 5.63		
	Non-ARNI	32.93 ± 9.48	41.67 ± 9.54	44.87 ± 9.99	48.87 ± 9.58		
	Group main effect					4.88	0.036*
	Time main effect					48.90	0*
	Group*Time					1.49	0.23

(mo:month;post-op:Post-operative).

## 4 Discussion

The present study investigated the potential risk factors for HFrEF developed from cardiac vavular surgery and presented with the short-term prognosis in such patients. Our results showed that about 8.1 % of the patients with preserved LVEF preoperatively developed HFrEF after cardiac valvular surgery, leading to higher mortality and increased consumption of medical resources. Among the numerous preoperative and intraoperative variables, our study found that only LVEDD was significantly related to HFrEF developed from surgery. Furthmore, we investigated the efficacy of sacubitril-valsartan in patients with surgery-related HFrEF and found that sacubitril valsartan was very effective in improving left ventricular remodeling and LVEF in such cohort.

As far as we know, factors such as an advanced age, the heart function grade, the left ventricular ejection fraction, diabetes, anemia, renal dysfunction, duration of the CPB and ascending aorta clamping were regarded as predictors of post-operative death or low cardiac output syndrome after cardiac surgery[5], [10], [11], [12]. Interestingly, none of these risk factors was found to be associated with the development of HFrEF after surgery. We reasoned the major difference lied in that the subjects of this study were patients with preserved LVEF before surgery and the average age in this cohort was relatively young. Secondly, although HFrEF significantly increased mortality and morbidity, the majority of the HFrEF patients survived and had a good quality of life. Lastly, statistical errors caused by an insufficient sample size may exist.

Our study showed that preoperative LVEDD was the only factor found to be significantly related to the development of HFrEF after cardiac valvular surgery. Since the range of normal LVEDD was rather large and different between male and female patients, we did not obtain the appropriate cut-off threshold for predicting post-operative HFrEF. Large LVEDD are reflected in a weakened left ventricular function reserve and decreased tolerance to ischemic reperfusion injury. Our results thus suggested that the timing of the surgical intervention should be considered when the left ventricle is enlarged in patients with valvular heart diseases, even if they had preserved LVEF.

The preoperative LVEF was 50 % or higher in all patients in the present study. Hence when HFrEF was diagnosed, their LVEF declined at least 20 % postoperatively. The exact mechanisms of the decreased myocardial function remain complex and yet unclear. The contributing factors include the preoperative myocardial dysfunction, ischemic reperfusion injury, the procedures of myocardial protection during operation, systemic inflammatory responses, and alterations in signal transduction systems[13], [14], [15]. Such dysfunctions might be temporary, mostly because of myocardial stunning, or can be persistent, due to a profound ischemia and myocardial infarction. Myocardial stunning is the condition in which the myocardium survives a transient ischemia reperfusion injury, but with its structure and metabolism changed. In this condition, myocardial function may be recovered hours, days, or even weeks after its reperfusion [16]. In order to minimize myocardial dysfunction, cardiac surgeons must observe extreme care regarding myocardial protection during surgery, by adopting a variety of strategies, including shortening the duration of the myocardial ischemia, the anterograde/retrograde myocardial perfusion with a modified cardioplegia solution, and maintaining the myocardial local cooling temperature [17], [18].

Undoubtedly, consistent arrthymia such as atrial fibrillation may contribute to further LVEF depression and was a potential confounding factor in this study. Irregular rapid rhythm may lead to inaccuracy in measuring LVEF and insufficient filling time for left ventricle, and anti-arrthymia drugs may further inhibit heart function. Therefore, although there was no difference in the rate of atrial fibrillation between the two groups, the role of arrthymia in this study was unclear.

We assumed that another contributing factor was the patient’s tolerance to ischemic reperfusion injury that was yet inadequately investigated. For instance, many studies have shown that female patients have better tolerance to ischemic reperfusion injury in the heart and other organs than male patients[19], [20]. Such protective effects could be related to sex hormones, including estrogen and testosterone, an hypothesis that has been verified in various animal models [21], [22]. In the present study, 67.6 % of the patients in the HFrEF group were male, which was significantly different from the non-HFrEF group (49.2 %). Although the gender was not statistically significant in the multivariate regression analysis, further investigation is needed.

In all 30 patients with surgery-related HFrEF, half received sacubitril-valsartan treatment at discharge and half not, which was at the discretion of their attending surgeon. As mentioned before, many studies demonstrated the positive effects of sacubitril/valsartan in patients with chronic heart failure with reduced ejection fraction (HFrEF). Abumayyaleh and colleagues showed that Sacubitril/Valsartan could continuously improve echocardiographic parameters including left ventricular LVEF, systolic pulmonary artery pressure, and cardiac valve insufficiency in HFrEF patients[23]. However, the efficacy and safety of sacubitril/valsartan in post-cardiac surgery related HFrEF patients remains unclear. For the concern of safety, the starting dosage of sacubitril-valsartan in this study was 25–50 mg twice a day and it was slowly titrated to 50–100 mg twice a day which was different in the common use of sacubitril-valsartan in chronic heart failure patients. However, even with such a low dosage, our study demonstrated that ARNI treatment was still very effective in improving LVEF and decreasing LVEDD and patients with ARNI treatment recovered more rapidly than those without ARNI treatment. Therefore, we recommend use of ARNI for patients developed HFrEF after cardiac surgery when there is no contraindications.

The present study has several limitations. Firstly, it was a single-center retrospective study. Secondly, although ejection fraction is a typical indicator of heart function, it is a dynamic parameter and could be affected by a number of factors such as heart rhythm, volume loading, valvular function, and vasoactive drugs. Thirdly, a post-operative LVEF ≤ 40 % as a criterium for HFrEF diagnosis is a rather arbitrary cut-off threshold. Post-operative LVEF might also decrease significantly in patients in the non-HFrEF group. Lastly, the study of the efficacy of sacubitril-valsartan treatment was non-random, the sample size was rather small, and effects of other anti-heart failure were possible confounding factors.

Funding.

This work was supported by a Natural Science Foundation of Hunan Province grant (2022JJ30929).

Authorship contribution statement:

Chen Yuqian(First author): Conceptualization, Methodology, Software, Investigation, Formal Analysis, Writing − Original Draft;

Hu Qinghua(Corresponding Author):Conceptualization, Funding Acquisition, Resources, Supervision, Writing − Review & Editing.

Luo Fanyan: Visualization, Investigation;

Huang lingjin: Resources, Supervision;

Chen Xuliang: Software, Validation;

Zhang Chengliang:Visualization, Writing − Review & Editing.

Ethics approval.

This study was approved and written informed consent was waived by the Ethics Committee of the Xiangya Hospital, Central South University (approval number: 202204103) due to it is retrospective nature. It was performed in compliance with the declaration of Helsinki.

## Declaration of competing interest

The authors declare that they have no known competing financial interests or personal relationships that could have appeared to influence the work reported in this paper.
